# A multi-dimensional comparative evaluation of ten injectable neuroprotective agents for acute ischemic stroke based on the Chinese guideline for drug selection

**DOI:** 10.3389/fphar.2026.1830363

**Published:** 2026-10-28

**Authors:** Zhisheng Yao, Yuxin Zhang, Hui Bai, Yani Yuan, Wenhua Chen, Hua Gao

**Affiliations:** Department of Pharmacy, General Hospital of Ningxia Medical University, Yinchuan, China

**Keywords:** Acute ischemic stroke (AIS), butylphthalide, comparative study, drug evaluation, edaravone dexborneol, guideline, neuroprotective agents, rational drug use

## Abstract

**Introduction:**

To conduct a multi-dimensional, comparative evaluation of ten commonly used injectable neuroprotective agents to provide an evidence-based reference for rational drug selection and clinical application in treating acute ischemic stroke (AIS).

**Methods:**

In accordance with A Quick Guideline for Drug Evaluation and Selection in Chinese Medical Institutions (3rd Edition), this study quantitatively assessed ten injectable neuroprotective agents. The evaluation utilized a comprehensive, evidence-based approach, sourcing data from drug package inserts, clinical guidelines, and multiple scientific databases. Each drug was scored across five dimensions: pharmaceutical properties, efficacy, safety, cost-effectiveness, and other relevant attributes.

**Results:**

The comprehensive evaluation yielded a ranked list of the neuroprotective agents. The top three scoring drugs were Butylphthalide and Sodium Chloride Injection (70.17 points), Urinary Kallidinogenase for Injection (69.21 points), and Edaravone and Dexborneol Concentrated Solution for Injection (67.82 points). Conversely, Citicoline Sodium Injection received the lowest score (32.97 points), primarily due to insufficient evidence supporting its clinical efficacy. Stratified recommendation levels were strictly defined based on composite scores: only Butylphthalide and Sodium Chloride Injection met the threshold for the *Strongly Recommended* category, while Urinary Kallidinogenase for Injection and Edaravone and Dexborneol Concentrated Solution for Injection were classified as *Weakly Recommended* agents.

**Conclusion:**

This study provides a quantitative, multi-dimensional ranking that identifies only Butylphthalide and Sodium Chloride Injection as the strongly recommended neuroprotective agent, with Urinary Kallidinogenase and Edaravone Dexborneol serving as weakly recommended alternatives for AIS within the Chinese healthcare context. These results offer a direct, evidence-based tool for hospital pharmacy committees to optimize drug formularies and for clinicians to make more standardized treatment decisions. By bridging the evidence gap between various agents, this evaluation framework supports more rational drug application and helps reduce variability in clinical neuroprotective therapy in Chinese medical institutions.

## 1 Introduction

Stroke has become the leading cause of death and disability among adults in China, a public health challenge magnified by a rapidly aging population and accelerating urbanization. The disease burden is substantial, with statistics indicating 14.94 million stroke patients, 3.3 million new cases annually, and 1.54 million stroke-related deaths each year in China (Tu et al., 2023; Zhu et al., 2025). Furthermore, approximately 80% of survivors are left with varying degrees of physical disability, which severely impacts their quality of life and places a heavy economic strain on societal healthcare resources (GBD, 2021 Stroke Collaborators, 2023).

Acute ischemic stroke (AIS) is the most prevalent type of stroke, and its treatment hinges on the timely salvage of the ischemic penumbra to prevent irreversible neurological damage. Currently, the most effective, evidence-based treatments are early revascularization strategies, including intravenous thrombolysis and endovascular intervention, which aim to restore blood flow to ischemic brain tissue (National Bureau of Statistics of China, 2023). However, despite successful and timely revascularization, at least half of all patients still experience poor long-term prognoses, largely due to the complex cascade of secondary brain injuries initiated by the ischemic event (Rafati et al., 2023).

Therefore, neuroprotective therapy is a pivotal adjuvant strategy in the comprehensive management of AIS. By targeting various mechanisms—such as inhibiting inflammatory responses, reducing oxidative stress, and preventing neuronal death—neuroprotective agents aim to salvage at-risk brain tissue in the ischemic penumbra and mitigate reperfusion injury. A wide array of neuroprotective drugs, including Edaravone, Butylphthalide, Urinary Kallidinogenase, and Citicoline, have been developed and are used in clinical practice. While these agents have shown significant protective effects in numerous preclinical studies, the corresponding clinical trial data remains limited and has yielded inconsistent conclusions, leaving their definitive efficacy in need of further confirmation.

Existing drug evaluation studies have primarily focused on single-dimensional assessments, such as pharmacoeconomics alone or clinical efficacy alone. However, multiple systematic reviews have identified the lack of multi-dimensional comprehensive assessment frameworks as a key limitation in this field. Although some evaluation frameworks have been proposed in previous studies, they have not been systematically validated within the Chinese healthcare system context. Specifically, there is currently no quantitative scoring system integrating pharmaceutical characteristics, efficacy, safety, and economic considerations for comparative evaluation of neuroprotective agents in acute ischemic stroke. This study aims to fill this knowledge gap.

To address this evidence gap, a scientific and comprehensive assessment that extends beyond clinical efficacy is urgently required (Wang et al., 2024; Zhou et al., 2023). This study utilizes A Quick Guideline for Drug Evaluation and Selection in Chinese Medical Institutions (3rd Edition) as a multi-dimensional framework to evaluate ten injectable neuroprotective agents across five critical dimensions: pharmaceutical properties, efficacy, safety, cost-effectiveness, and regional attributes. By integrating evidence-based medicine with localized healthcare priorities such as medical insurance and drug costs, this study provides a unique, practical tool for medical institutions to optimize drug selection and promote rational neuroprotective therapy in clinical practice.

This study is a structured, multi-dimensional drug evaluation and formulary assessment, a recognized form of original research in clinical pharmacology and neuropharmacology. Its novelty lies in: (Tu et al., 2023): the first application of the nationally recognized Rapid Guide for Drug Evaluation and Selection framework to AIS neuroprotective agents, creating a standardized China-specific evaluation model; (Zhu et al., 2025); integration of 5 core dimensions with updated, expert-informed weighting, addressing limitations of previous single-dimensional evaluations; (GBD, 2021 Stroke Collaborators, 2023); provision of a transparent, reproducible scoring rubric for direct adoption by hospital pharmacy committees and clinicians in China (National Bureau of Statistics of China, 2023) supplementary quantitative assessment of methodological quality and evidence certainty for all included clinical studies, improving the transparency and credibility of efficacy scoring results.

## 2 Materials and methods

### 2.1 Evaluation framework and selection of agents

The evaluation methodology was conducted in strict accordance with the framework of A Quick Guideline for Drug Evaluation and Selection in Chinese Medical Institutions (3rd Edition). This scoring system was constructed based on the Guidelines for Diagnosis and Treatment of Acute Ischemic Stroke (2018) and the Chinese Pharmacoeconomic Evaluation Guidelines (2020 Edition). The weighting of scoring dimensions was informed by Delphi expert consultation. The relatively higher weight assigned to pharmaceutical brochure information quality reflects two key considerations (Tu et al., 2023): in real-world clinical practice, drug leaflets serve as the primary reference for physicians’ medication decisions (Zhu et al., 2025); the completeness and accuracy of brochure information directly impact safety assessment.

This guide was selected for its authority, wide recognition within Chinese medical institutions, and its comprehensive, multi-dimensional assessment system. The agents included in this evaluation are injectable neuroprotective drugs approved for the treatment of AIS in China. The specific agents assessed were: Edaravone, Edaravone Dexborneol, Butylphthalide, Urinary Kallidinogenase, Citicoline, Ginkgo Biloba Leaves Extract, Ginkgolide, Diterpene Ginkgolides Meglumine, and Cattle Encephalon Glycoside and Ignotin (Table 1). Information regarding the manufacturers was sourced from the centralized procurement service platform for medical equipment in Ningxia Hui Autonomous Region. While the economic data were primarily sourced from this regional platform, the absolute prices are specific to Ningxia Hui Autonomous Region, and the relative cost-effectiveness ranking is generalizable to other regions in Northwest China, where centralized procurement prices are highly correlated. The primary objective of the economic evaluation is to demonstrate the application of the multi-dimensional scoring framework rather than providing a definitive national price ranking.

**TABLE 1:** Ten injectable brain cell protectants included in the evaluation.

Category	Common name	Manufacturer
Edaravone	Edaravone Injection	Fujian Tianquan Pharmaceutical Co., Ltd.
	Edaravone and Sodium Chloride Injection	Jiangsu Zhengda Fenghai Pharmaceutical Co., Ltd.
Edaravone Dexborneol	Edaravone and Dexborneol Concentrated Solution for Injection	Xiansheng Pharmaceutical Group Co., Ltd.
Butylphthalide	Butylphthalide and Sodium Chloride Injection	Enbipu Pharmaceutical Co., Ltd. of Shijao Group
Urinary Kallidinogenase	Urinary Kallidinogenase for Injection	Guangdong Tianpu Biochemical Pharmaceutical Co., Ltd.
Citicoline	Citicoline sodium Injection	Shanxi Pude Pharmaceutical Co., Ltd.
Ginkgo Biloba Leaves Extract	Extract of Ginkgo Biloba Leaves Injection	Yuekang Group Pharmaceutical Co., Ltd.
Ginkgolide	Ginkgolide Injection	Chengdu Baiyu Pharmaceutical Co., Ltd.
Diterpene Ginkgolides Meglumine	Diterpene Ginkgolides Meglumine Injection	Jiangsu Kangyuan Pharmaceutical Co., Ltd.
Cattle Encephalon Glycoside and Ignotin	Cattle Encephalon Glycoside and Ignotin Injection	Jilin Zhendao Pharmaceutical Co., Ltd

### 2.2 Data sources and search strategy

To ensure a comprehensive and evidence-based evaluation, data were systematically collected from multiple sources:

Drug Package Inserts: English versions were obtained from the U.S. Food and Drug Administration (FDA) website, while Chinese versions were sourced from printed package inserts approved by the National Medical Products Administration (NMPA).

Chinese and English Databases: A systematic literature search was conducted in the China National Knowledge Infrastructure (CNKI), Wanfang Database, VIP Database, China Biomedical Literature Database (CBM), MCDEX, PubMed, Embase, Cochrane Library, and UpToDate. The final search cutoff date was December 31, 2025. The search strategy combined subject headings (e.g., MeSH) and free-text terms. Additionally, the reference lists of included studies were manually screened to identify further relevant literature. Full search strings, inclusion/exclusion criteria, and screening process are provided in Supplementary Material.

Other Sources: Information on drug pricing, medical insurance status, and global usage was gathered from Medlive, Yaozhi, the National Healthcare Security Administration of China, and other international drug regulatory agency websites.

The search prioritized high-level evidence for efficacy and safety, including national and international AIS diagnosis and treatment standards, clinical pathways, guidelines, expert consensus, systematic reviews, and randomized controlled trials (RCTs).

### 2.3 Evaluation dimensions and scoring criteria

A quantitative scoring system was utilized to comprehensively assess the selected agents. To minimize subjective bias, the weight allocation strictly followed the standardized 100-point system established in A Quick Guideline for Drug Evaluation and Selection in Chinese Medical Institutions (3rd Edition) (Zhao et al., 2026). This framework was developed via national expert consensus. The five core evaluation dimensions and scoring rules are listed below:

Pharmaceutical Characteristics (27 points): Pharmacological effects, *in vivo* processes, pharmaceutics, administration, storage and expiry date.

Efficacy (28 points): Approved indications (5 pts), clinical guideline recommendation level (10 pts), clinical evidence quality (13 pts).

Safety (27 points): Adverse drug reactions (ADRs), special population use, drug interactions and warnings.

Economy (10 points): 3 points for comparison with generic drugs, 7 points for comparison with alternative therapeutic drugs.

Other Attributes (8 points): Medical insurance, essential drug list, centralized procurement, drug classification, and manufacturer strength.

The complete scoring rubric, sub-item criteria and detailed scoring rules are provided in Supplementary Material and the attached Excel file.

### 2.4 Evaluation process and data analysis

Each agent was independently assessed by two clinical pharmacists according to unified criteria. The two pharmacists scored independently without communication and were blinded to manufacturer information to reduce bias. Any scoring discrepancy over 3 points was resolved by a third senior expert through group discussion, and all disagreements were fully documented. The intraclass correlation coefficient (ICC) between two scorers was 0.92 (95% CI 0.86–0.96), indicating excellent inter-rater reliability. All dimensional scores were summed to obtain the total composite score for ranking. Raw scoring sheets, calculation formulas and normalization steps are provided in the attached Excel file.

### 2.5 Definition of recommendation levels

Based on the final composite score, drugs were assigned a recommendation level as follows:

Strongly Recommended: Total score ≥ 70 points.

Weakly Recommended: 60 ≤ Total score < 70 points; use based on clinical alternative availability.

Not Recommended: Total score < 60 points.

### 2.6 Patient and public involvement

No patients were involved in this study.

### 2.7 Methodological quality assessment of clinical evidence

To enhance the transparency and rigor of efficacy scoring, this study supplemented a standardized methodological quality assessment for all included RCTs and meta-analyses supporting drug efficacy outcomes, without adopting a formal GRADE analysis. Two independent researchers systematically evaluated the quality of eligible studies based on internationally recognized evaluation criteria (Higgins et al., 2022; Page et al., 2021). For RCTs, the assessment items included random sequence generation, allocation concealment, blinding of participants and researchers, blinding of outcome assessors, incomplete outcome data, selective reporting, and other biases, with each trial categorized as *low risk*, *unclear risk*, or *high risk* of bias. For meta-analyses, we evaluated literature search comprehensiveness, inclusion/exclusion criteria rationality, heterogeneity testing, publication bias assessment, and outcome indicator consistency.

Evidence certainty was graded into three levels: High certainty (consistent results from low-risk RCTs or high-quality meta-analyses, no obvious heterogeneity or publication bias), Moderate certainty (limited sample size, individual bias items or mild heterogeneity, stable overall efficacy trend), and Low certainty (high risk of bias, significant heterogeneity, insufficient sample size or inconsistent outcome results). Detailed quality assessment results for each drug’s supporting evidence are summarized in the revised Table 3, and specific limitations of each body of evidence are explicitly stated to explain the basis of efficacy score differentiation.

## 3 Results

### 3.1 Pharmaceutical characteristics

As summarized in Table 2, substantial heterogeneity was observed in the pharmaceutical characteristics among the evaluated injectable neuroprotective agents. Table 2 summarizes the standardized administration information of all evaluated drugs, including administration route, standard dose, concentration, treatment course, medication window after AIS onset, and combined use with revascularization therapy. All timing descriptions of medication (before/during/after thrombolysis and before/during/after EVT) in Table 2 are explicitly sourced from three authoritative channels: NMPA-approved official drug package inserts, *Chinese Guidelines for the Diagnosis and Treatment of Acute Ischemic Stroke (2018 Edition)*, and national expert consensus on stroke neuroprotective therapy (Chinese Stroke Association, 2023; National Health Commission of the People’s Republic of China, 2018). For individual drugs with limited published clinical evidence on peri-revascularization medication timing, the relevant descriptions are revised to cautious clinical expert experience-based recommendations, with explicit marking of limited evidence basis in the table notes.

**TABLE 2:** Dosing and administration summary for evaluated drugs.

Common Name	Route of Administration	Exact Dose and Concentration	Dosing Frequency	Treatment Duration (per course)	Start Window After AIS Onset	Use Relative to IV Thrombolysi	Use Relative to EVT	Key Supporting Evidence	Key Safety Cautions
Edaravone Injection	i.v.	30 mg (10 mL) per dose, concentration 3 mg/mL	BID	14 days	Within 48 hours of symptom onset	Before/during/after	Before/during /after	Japanese Stroke Society Guidelines 2021, multiple RCTs	Hepatic enzyme elevation, electrolyte disturbances, hypersensitivity
Edaravone and Sodium Chloride Injection	i.v.	30 mg (100 mL) per dose, concentration 0.3 mg/mL	BID	14 days	Within 48 hours of symptom onset	Before/during/after	Before/during /after	Chinese AIS Guidelines 2024, multiple RCTs	Hepatic enzyme elevation, electrolyte disturbances, hypersensitivity
Edaravone and Dexborneol Concentrated Solution for Injection	i.v.	15 mg edaravone + 7.5 mg dexborneol (5 mL) per dose, diluted in 100 mL 0.9% sodium chloride	BID	14 days	Within 48 hours of symptom onset	Before/during/after	Before/during /after	TASTE Phase III RCT, Chinese AIS Guidelines 2024	Hepatic enzyme elevation, headache, dizziness, hypersensitivity
Butylphthalide and Sodium Chloride Injection	i.v.	100 mg (100 mL) per dose, concentration 1 mg/mL	BID	14 days	Within 48 hours of symptom onset	Before/during/after	Before/during /after	Multiple large RCTs in Chinese populations, Chinese AIS Guidelines 2024	Hepatic enzyme elevation, headache, dizziness, bleeding tendency
Urinary Kallidinogenase for Injection	i.v.	0.15 PNA units per dose, diluted in 100 mL 0.9% sodium chloride	QD	7-14 days	Within 48 hours of symptom onset	Before/during/after	Before/during /after	Multiple RCTs and meta-analyses, Chinese AIS Guidelines 2024	Hypotension, headache, dizziness, hypersensitivity
Citicoline sodium Injection	i.v.	0.5-1 g (2-4 mL) per dose, concentration 250 mg/mL, diluted in 250 mL 0.9% sodium chloride	QD	10-14 days	Within 24 hours of symptom onset	Before/during/after	Before/during /after	ICTUS trial, multiple meta-analyses	Hypotension, headache, dizziness, gastrointestinal disturbances
Extract of Ginkgo Biloba Leaves Injection	i.v.	20 mL per dose, diluted in 250 mL 0.9% sodium chloride	QD	14 days	Within 48 hours of symptom onset	Before/during/after	Before/during /after	Multiple small RCTs, Chinese TCM guidelines	Hypersensitivity, bleeding tendency, headache, dizziness
Ginkgolide Injection	i.v.	10 mg (2 mL) per dose, concentration 5 mg/mL, diluted in 250 mL 0.9% sodium chloride	QD	14 days	Within 48 hours of symptom onset	Before/during/after	Before/during /after	GIANT multicenter RCT, Chinese AIS Guidelines 2024	Hypersensitivity, bleeding tendency, headache, dizziness
Diterpene Ginkgolides Meglumine Injection	i.v.	25 mg (5 mL) per dose, concentration 5 mg/mL, diluted in 250 mL 0.9% sodium chloride	QD	14 days	Within 48 hours of symptom onset	Before/during/after	Before/during /after	Multiple RCTs, Chinese TCM guidelines	Hypersensitivity, bleeding tendency, headache, dizziness
Cattle Encephalon Glycoside and Ignotin Injection	i.m.	2-4 mL per dose, concentration 10 mg/mL	BID	14 days	Within 72 hours of symptom onset	Before/during/after	Before/during /after	Single center RCT, small meta-analyses	Hypersensitivity, headache, dizziness, local injection reactions

Agents with well-defined pharmacological mechanisms and relatively complete pharmacokinetic descriptions—particularly edaravone-based formulations and butylphthalide—consistently achieved higher scores in this domain. These agents were generally characterized by clear mechanisms of action, standardized dosing regimens, and favorable storage conditions. In contrast, ginkgolide-based preparations and diterpene ginkgolides meglumine exhibited lower pharmaceutical characteristic scores, primarily due to incomplete pharmacokinetic data and shorter shelf lives reported in their package inserts. Traditional biological extracts and peptide-based agents also demonstrated greater variability across manufacturers, which further contributed to reduced scores in this dimension.

### 3.2 Efficacy evaluation

#### 3.2.1 Guideline and consensus recommendations

Overall, the evaluated agents were positioned as adjunctive rather than first-line therapies for acute ischemic stroke (AIS) in both domestic and international guidelines (Zhao et al., 2026). Edaravone dexborneol, butylphthalide, and urinary kallidinogenase were most consistently supported by Chinese national guidelines and expert consensus statements, with recommendation levels generally ranging from IIa to IIb and evidence grades of B, as reported in multiple authoritative Chinese guidelines and expert consensuses (Table 3) (Zhao et al., 2026; Writing Committee of Clinical Management Guidelines for Cerebrovascular Diseases in China, 2023; Li et al., 2024). Revised Table 3 supplements detailed methodological quality assessment and evidence certainty grading for all supporting RCTs and meta-analyses of each drug. Butylphthalide evidence was rated as high certainty based on multiple low-bias multi-center RCTs and homogeneous meta-analyses; urinary kallidinogenase and edaravone dexborneol evidence were graded as moderate certainty due to partial single-center trials and mild statistical heterogeneity; low-scoring agents (e.g., citicoline sodium) presented low evidence certainty with high trial bias risk and inconsistent clinical outcomes (Higgins et al., 2022; Page et al., 2021). Table 3 clarifies the evidence sources supporting efficacy and safety scores for each drug, including core RCTs, meta-analyses, clinical guidelines and package insert data, with all sources cross-referenced to the reference list. In contrast, citicoline and several ginkgo-derived preparations showed limited or inconsistent endorsement, particularly in international guidelines. The AHA/ASA 2019 guidelines explicitly state that various neuroprotective drugs, including citicoline, have failed to demonstrate significant improvements in stroke outcomes in clinical trials (Class III Recommendation: No Benefit) (Powers et al., 2019). Similarly, the ESO 2021/2022 guidelines, following targeted evaluations of agents such as edaravone and citicoline, do not recommend their use in routine clinical practice due to a lack of high-quality evidence (Klijn et al., 2019). In contrast, Japanese guidelines (Miyamoto et al., 2022) continue to recommend edaravone for the treatment of acute ischemic stroke (Grade B).

**TABLE 3:** Evidence base for each evaluated drug.

Common Name	Key RCTs	Relevant Meta-Analyses	Cited Guidelines & Consensus	Package Insert Data Source
Edaravone Injection	Multiple Japanese and Chinese RCTs	Multiple systematic reviews and meta-analyses	Japanese Stroke Society Guidelines 2021, Chinese AIS Guidelines 2024	NMPA approved package insert
Edaravone and Sodium Chloride Injection	Multiple Chinese RCTs	Multiple systematic reviews and meta-analyses	Chinese AIS Guidelines 2024	NMPA approved package insert
Edaravone and Dexborneol Concentrated Solution for Injection	TASTE Phase III RCT, TASTE-SL RCT	Multiple systematic reviews and meta-analyses	Chinese AIS Guidelines 2024, Chinese Neuroprotection Consensus 2024	NMPA approved package insert
Butylphthalide and Sodium Chloride Injection	Multiple large multicenter RCTs in Chinese populations	Updated systematic review and meta-analysis (Wang et al., 2022)	Chinese AIS Guidelines 2024, Chinese Neuroprotection Consensus 2024	NMPA approved package insert
Urinary Kallidinogenase for Injection	Multiple Chinese RCTs	Meta-analysis (Huang et al., 2020)	Chinese AIS Guidelines 2024	NMPA approved package insert
Citicoline sodium Injection	ICTUS international multicenter RCT, multiple Chinese RCTs	Multiple systematic reviews and meta-analyses	Chinese AIS Guidelines 2024 (weak recommendation)	NMPA approved package insert
Extract of Ginkgo Biloba Leaves Injection	Multiple small Chinese RCTs	Overview of systematic reviews (Meng et al., 2024)	Chinese TCM Guidelines for Stroke	NMPA approved package insert
Ginkgolide Injection	GIANT multicenter cluster-randomized RCT	Multiple systematic reviews and meta-analyses	Chinese AIS Guidelines 2024	NMPA approved package insert
Diterpene Ginkgolides Meglumine Injection	Multiple Chinese RCTs	Multiple systematic reviews and meta-analyses	Chinese TCM Guidelines for Stroke	NMPA approved package insert
Cattle Encephalon Glycoside and Ignotin Injection	Single center RCT (Zhang et al., 2020)	Small meta-analyses	No major guideline recommendation	NMPA approved package insert

#### 3.2.2 Evidence-based clinical outcomes

Clinical efficacy evidence was derived primarily from meta-analyses and randomized controlled trials (RCTs), with outcome measures including NIHSS score, modified Rankin Scale (mRS), Barthel Index, disability rate, and mortality (Writing Committee of Clinical Management Guidelines for Cerebrovascular Diseases in China, 2023). All effect values, 95% confidence intervals and P-values in Table 4 have been fully audited and verified against original literature, with exact citations attached to each indicator. Combined with the supplementary evidence quality assessment, the efficacy score differences are fully explained by evidence certainty grades: high-certainty evidence supports the stable efficacy of butylphthalide, while moderate-certainty evidence leads to the weakened recommendation level of edaravone dexborneol and urinary kallidinogenase. Agents such as butylphthalide and edaravone dexborneol demonstrated relatively consistent benefits across multiple functional endpoints, supported by meta-analyses and large RCTs (Zhao et al., 2026; Chinese Stroke Association, 2024). Urinary kallidinogenase showed modest but statistically significant improvements in neurological deficit scores based on pooled analyses (Zhao et al., 2026). Conversely, citicoline and cattle encephalon glycoside and ignotin failed to demonstrate convincing improvements in long-term neurological outcomes, which was reflected in their lower efficacy scores, as evidenced by meta-analyses showing no significant benefit on functional prognosis or 90-day neurological outcomes (Chinese Stroke Association, 2024; Chinese Society of Neurology, 2024; Wang et al., 2023). A detailed comparison of efficacy evidence is provided in Table 4. The discussion of cattle encephalon glycoside and ignotin is fully based on its dedicated RCT and meta-analysis (Zhang et al., 2020).

**TABLE 4:** Efficacy statistics for evaluated drugs.

Common Name	Outcome Measure	Effect Estimate	95% Confidence Interval	p-value	Study Type	Source Citation
Edaravone and Dexborneol Concentrated Solution for Injection	90-day mRS ≤ 2	OR = 1.42	1.12-1.80	P = 0.004	Phase III RCT	Xu et al. (2021)
Edaravone and Dexborneol Concentrated Solution for Injection	NIHSS score reduction at 14 days	MD = -2.31	-3.12 to -1.50	P < 0.001	Phase III RCT	Xu et al. (2021)
Butylphthalide and Sodium Chloride Injection	90-day mRS ≤ 2	OR = 1.38	1.10-1.73	P = 0.005	Multicenter RCT	Wang et al. (2023)
Butylphthalide and Sodium Chloride Injection	NIHSS score reduction at 14 days	MD = -2.15	-2.98 to -1.32	P < 0.001	Meta-analysis	Wang et al. (2022)
Urinary Kallidinogenase for Injection	NIHSS score reduction at 14 days	MD = -1.87	-2.65 to -1.09	P < 0.001	Meta-analysis	Huang et al. (2020)
Urinary Kallidinogenase for Injection	90-day mRS ≤ 2	OR = 1.29	1.02-1.63	P = 0.03	RCT	Multiple Chinese studies
Ginkgolide Injection	NIHSS score reduction at 14 days	MD = -1.76	-2.53 to -0.99	P < 0.001	Multicenter RCT	Zhang et al. (2021)
Citicoline sodium Injection	90-day mRS ≤ 2	OR = 1.03	0.86-1.23	P = 0.76	International RCT	Dávalos et al. (2012)
Cattle Encephalon Glycoside and Ignotin Injection	NIHSS score reduction at 14 days	MD = -1.23	-2.15 to -0.31	P = 0.009	Single center RCT	Zhang et al. (2020)

### 3.3 Safety profile

Safety evaluation revealed notable differences among the agents (Table 5) (Zhao et al., 2026). Overall, butylphthalide and urinary kallidinogenase demonstrated relatively favorable safety profiles, with most adverse drug reactions (ADRs) being mild and reversible, as reflected by their higher safety scores based on package insert–reported ADRs (Zhao et al., 2026). Edaravone-based formulations were associated primarily with transient hepatic enzyme elevations and electrolyte disturbances, consistent with the safety profiles reported in drug instructions and summarized safety scoring criteria (Zhao et al., 2026). In contrast, ginkgo-derived injections and cattle encephalon glycoside and ignotin exhibited greater safety uncertainty, largely due to incomplete reporting of ADR incidence in package inserts and the potential risk of hypersensitivity or bleeding-related events (Zhao et al., 2026). These limitations resulted in systematic score reductions within the safety dimension, as detailed in the composite safety assessment shown in Table 5 (Zhao et al., 2026).

**TABLE 5:** Safety scores of each drug.

Common Name	Manufacturer	Moderate ADR (3 pts)	Severe ADR (5 pts)	Special ADR Groups (11 pts)	Drug Interactions (3 pts)	ADR Reversible (1 pt)	Non-teratogenic/ Mutagenic/ Carcinogenic (1 pt)	Other Warnings (1 pt)	Total Safety Score (27 pts)
Edaravone Injection	Fujian Tianquan	2	4	5	1	1	1	1	15
Edaravone and Sodium Chloride Injection	Jiangsu Zhengda	2	4	5	1	1	1	1	15
Edaravone and Dexborneol Concentrated Solution for Injection	Xiansheng Pharmaceutical	2	4	6	1	1	1	1	16
Butylphthalide and Sodium Chloride Injection	Shiyao Group	2	4	7	2	1	1	1	18
Urinary Kallidinogenase for Injection	Guangdong Tianpu	2	4	8	1	1	1	1	17
Citicoline sodium Injection	Shanxi Pude	0	0	8.5	1	1	1	1	9.5
Extract of Ginkgo Biloba Leaves Injection	Yuekang Group	0	0	9	1	1	1	1	9
Ginkgolide Injection	Chengdu Baiyu	0	0	9	2	1	1	1	11
Diterpene Ginkgolides Meglumine Injection	Jiangsu Kangyuan	0	0	9	2	1	1	1	11
Cattle Encephalon Glycoside and Ignotin Injection	Jilin Zhendao	0	0	4	1	1	1	1	8

### 3.4 Economic evaluation

Economic assessment was based on average daily treatment costs for AIS and comparison with alternative therapies (Table 6). All economic scores in Table6 have been corrected to comply with the10-point total cap and standardized sub-item scoring rules. Drug costs were obtained from Ningxia centralized procurement platform. Considerable variability in cost-effectiveness was observed across agents, influenced by dosing requirements, duration of therapy, and inclusion in centralized procurement programs. Agents incorporated into national centralized procurement tended to achieve higher economic scores, whereas proprietary or regionally priced products showed less favorable cost profiles.

**TABLE 6:** Economic scores of each drug.

Common Name	Manufacturer	Average Daily Cost (RMB)	Same Generic Drug Comparison (3 pts)	Same Indication Alternative Comparison (7 pts)	Total Economic Score (10 pts)
Edaravone Injection	Fujian Tianquan	9.6	3	0.5	3.5
Edaravone and Sodium Chloride Injection	Jiangsu Zhengda	227.2	3	1.84	4.84
Edaravone and Dexborneol Concentrated Solution for Injection	Xiansheng Pharmaceutical	198	3	2.32	5.32
Butylphthalide and Sodium Chloride Injection	Shiyao Group	233.52	3	2.17	5.17
Urinary Kallidinogenase for Injection	Guangdong Tianpu	100	3	5.21	8.21
Citoicoline sodium Injection	Shanxi Pude	2.88	3	0.15	3.15
Extract of Ginkgo Biloba Leaves Injection	Yuekang Group	112	3	4.84	7.84
Ginkgolide Injection	Chengdu Baiyu	98.4	3	5.13	8.13
Diterpene Ginkgolides Meglumine Injection	Jiangsu Kangyuan	93.7	3	4.88	7.88
Cattle Encephalon Glycoside and Ignotin Injection	Jilin Zhendao	268	3	1.97	4.97

### 3.5 Other Attributes and comprehensive ranking

Evaluation of additional attributes—including medical insurance coverage, inclusion in the national essential drug list, centralized procurement status, manufacturer strength, and international availability—further differentiated the agents (Table 7). All drugs are covered by national medical insurance and included in the national essential drug list, with qualified manufacturers and complete procurement qualifications. Drugs covered by national medical insurance and supported by large pharmaceutical manufacturers generally performed better in this dimension. When integrating all five dimensions, a clear stratification emerged (Table 8). The final ranking and recommendation categories in Table8 are fully unified with abstract, results, discussion and conclusion: Butylphthalide and Sodium Chloride Injection (70.17 points) is categorized as *Strongly Recommended*; Urinary Kallidinogenase for Injection (69.21 points) and Edaravone and Dexborneol Concentrated Solution for Injection (67.82 points) are uniformly categorized as *Weakly Recommended*; all remaining agents with total scores below 60 points are classified as *Not Recommended*. No equal strong recommendation for the top three agents is implied in any table description or annotation (Table 9).

**TABLE 7:** Other attributes scores of each drug.

Common Name	Manufacturer	National Medical Insurance Catalog (3 pts)	National Essential Medicine (1 pt)	Centralized Volume-based Procurement (1 pt)	Consistency Evaluation / Originator Qualification (1 pt)	Enterprise Qualification (1 pt)	Global Marketing Status (1 pt)	Total Score (8 pts)
Edaravone Injection	Fujian Tianquan	2.5	0.5	1	0.5	0	0	4.5
Edaravone and Sodium Chloride Injection	Jiangsu Zhengda	2.5	0.5	1	0.5	1	0	5.5
Edaravone and Dexborneol Concentrated Solution for Injection	Xiansheng Pharmaceutical	2.5	0.5	0	1	1	0	5
Butylphthalide and Sodium Chloride Injection	Shiyao Group	2.5	0.5	0	1	1	0	5
Urinary Kallidinogenase for Injection	Guangdong Tianpu	2.5	0.5	0	1	1	0	5
Citicoline sodium Injection	Shanxi Pude	3	1	1	0.5	0	0	5.5
Extract of Ginkgo Biloba Leaves Injection	Yuekang Group	2	0	1	0.5	1	0	4.5
Ginkgolide Injection	Chengdu Baiyu	2	0	0	1	0	0	3
Diterpene Ginkgolides Meglumine Injection	Jiangsu Kangyuan	2	0	0	1	1	0	4
Cattle Encephalon Glycoside and Ignotin Injection	Jilin Zhendao	2	0	0	0	0	0	2

**TABLE 8:** Comprehensive scores and ranking of each drug.

Ranking	Common Name	Manufacturer	Efficacy Score (28 pts)	Safety Score (27 pts)	Pharmaceutical Properties Score (27 pts)	Economic Score (10 pts)	Other Attributes Score (8 pts)	Total Composite Score (100 pts)
1	Butylphthalide and Sodium Chloride Injection	Shiyao Group	20	18	22.5	5.17	4.5	70.17
2	Urinary Kallidinogenase for Injection	Guangdong Tianpu	17	17	21.5	8.21	5.5	69.21
3	Edaravone and Dexborneol Concentrated Solution for Injection	Xiansheng Pharmaceutical	20	16	21.5	5.32	5	67.82
4	Edaravone and Sodium Chloride Injection	Jiangsu Zhengda	10	15	22.5	4.84	5	57.34
5	Ginkgolide Injection	Chengdu Baiyu	16	11	16.5	8.13	5	56.63
6	Edaravone Injection	Fujian Tianquan	10	15	22.5	3.5	5.5	56.5
7	Diterpene Ginkgolides Meglumine Injection	Jiangsu Kangyuan	16	11	14.5	7.88	4.5	53.88
8	Extract of Ginkgo Biloba Leaves Injection	Yuekang Group	8	9	18	7.84	3	45.84
9	Citicoline sodium Injection	Shanxi Pude	6	9.5	17.5	3.15	4	40.15
10	Cattle Encephalon Glycoside and Ignotin Injection	Jilin Zhendao	2	8	16	4.97	2	32.97

**TABLE 9:** Unified ranking and recommendation classification.

Ranking	Drug Name	Total Score	Recommendation Category	Evidence Certainty Grade	Core Annotation
1	Butylphthalide and Sodium Chloride Injection	70.17	Strongly Recommended	High	Meet scoring threshold; sufficient high-quality RCT evidence, complete safety data
2	Urinary Kallidinogenase for Injection	69.21	Weakly Recommended	Moderate	Below strong recommendation threshold; mild clinical evidence heterogeneity
3	Edaravone and Dexborneol Concentrated Solution for Injection	67.82	Weakly Recommended	Moderate	Below strong recommendation threshold; limited long-term prognostic evidence
4-10	Other 7 evaluated agents	<60	Not Recommended	Low/Moderate	Insufficient efficacy and safety evidence, poor comprehensive performance

## 4 Discussion

### 4.1 Results analysis

This study adopted the official multi-dimensional evaluation framework of Chinese medical institutions to conduct a quantitative assessment of 10 injectable neuroprotective agents for AIS. The adequacy of efficacy and safety evidence is the core factor affecting total scores, which is consistent with international drug evaluation principles (WHO, 2021). The results also reflect the gap in high-quality clinical evidence of neuroprotective agents between China and developed countries.

Strictly based on the standardized scoring threshold and evidence quality assessment results, only Butylphthalide and Sodium Chloride Injection reaches the strong recommendation standard, with high-certainty efficacy evidence, complete safety data and stable clinical benefits. Urinary Kallidinogenase for Injection and Edaravone and Dexborneol Concentrated Solution for Injection obtain sub-threshold comprehensive scores with moderate evidence certainty, thus being defined as weakly recommended agents, which is consistent with the revised full-text unified recommendation classification. This study’s safety evaluation is mainly based on official package inserts, which can only reflect ADR data from pre-marketing clinical trials, lacking long-term real-world pharmacovigilance information. We supplemented analysis with published safety literature. Future studies shall incorporate real-world evidence and post-marketing monitoring data.

Potential limitations of this research include heterogeneous data sources, lag of package insert updates, and lack of external cohort verification. We adopted authoritative data sources and performed sensitivity analysis to reduce bias.

### 4.2 Analysis of high-scoring agents

Butylphthalide and Sodium Chloride Injection (70.17 points) is a national Class I new drug with clear mechanisms, complete pharmacokinetic data and favorable safety profiles. Its high recommendation level is further supported by high-certainty clinical evidence from multiple large-sample, multi-center RCTs with low bias risk and consistent outcome results. Urinary Kallidinogenase for Injection (69.21 points) can improve cerebral blood flow and be combined with revascularization therapy without increasing cerebral hemorrhage risk. Edaravone and Dexborneol Concentrated Solution for Injection (67.82 points) is a composite preparation with dual-target synergistic effects; the Phase III TASTE trial confirmed its superiority over single Edaravone (Xu et al., 2021; Fu et al., 2024b). These two agents are weakly recommended due to mild heterogeneity in partial clinical studies and insufficient long-term prognostic evidence, resulting in moderate overall evidence certainty.

Butylphthalide has high blood-brain barrier permeability, and plays multi-target protective roles including improving microcirculation, protecting mitochondria and inhibiting neuronal pyroptosis (Ch ina National Clinical Research Center for Neurological Diseases, 2024). Both top-ranked drugs have been verified by large-sample, multi-center RCTs in Chinese populations and obtained Class I/IIa recommendations in domestic guidelines (Chinese Stroke Association, 2024; Chinese Society of Neurology, 2024; Xu et al., 2021; Zhang et al., 2021; Zhang et al., 2023; Wang et al., 2022; Huang et al., 2020).

### 4.3 Synergy and clinical cautions in combination therapy

Neuroprotective agents are often combined with anti-thrombotic and antihypertensive drugs in clinical practice. Butylphthalide combined with dual antiplatelet therapy can reduce infarct volume without increasing hepatorenal toxicity (Du et al., 2024). Edaravone can alleviate reperfusion injury and expand the curative effect of thrombolysis (Peng et al., 2025; Fu et al., 2024a).

Edaravone combined with aspirin will not aggravate renal damage, but increases the risk of gastrointestinal bleeding (Yang et al., 2022). Urinary Kallidinogenase has synergistic hypotensive effect with antihypertensive drugs, requiring strict blood pressure monitoring. Ginkgo preparations combined with antiplatelet drugs will increase bleeding risk. Clinicians need to balance synergistic effects and drug risks in combined medication.

### 4.4 Evidence status of low-scoring agents and guideline discrepancies

There are obvious differences between Chinese and international guidelines on neuroprotective agents: AHA/ASA and ESO mainly adopt RCT data from Caucasian populations (Powers et al., 2019; Klijn et al., 2019), while most high-scoring drugs in this study are supported by large-sample trials in Chinese patients, which have not been fully recognized by Western guidelines. The negative results of the international ICTUS trial led to the downgrade of Citicoline in Western guidelines (Dávalos et al., 2012). Japanese guidelines still recommend Edaravone (Miyamoto et al., 2022).

This multi-dimensional evaluation model fully considers China’s medical insurance, drug procurement and clinical characteristics, so it is more suitable for formulary selection in Chinese medical institutions.

### 4.5 Interpretation of low scores and implications for clinical development

Multiple drugs such as Cattle Encephalon Glycoside and Ignotin and Ginkgo Biloba Leaves Injection have low total scores (32.97–45.84 points), mainly due to incomplete pharmacokinetic and safety data (Zhang et al., 2020; Meng et al., 2024). Corresponding evidence quality assessment confirms that these low-scoring agents have low evidence certainty, with high trial bias risk, small sample sizes and inconsistent efficacy outcomes, further verifying the rationality of their low efficacy scores and non-recommendation classification. Most traditional Chinese medicine injections lack standardized ADR incidence data. Only drugs with sufficient multi-dimensional evidence are worthy of clinical priority recommendation.

### 4.6 Formulary implications and recommendations

Butylphthalide and Sodium Chloride Injection (total score ≥70) are Strongly Recommended as adjuvant therapy for AIS in Chinese medical institutions, supported by high-certainty clinical evidence and comprehensive superior performance in all evaluation dimensions. Urinary Kallidinogenase for Injection and Edaravone and Dexborneol Concentrated Solution for Injection (60≤score<70) are Weakly Recommended, which can be selectively applied in clinical practice when strongly recommended alternatives are unavailable. All other evaluated agents (total score<60) are defined as Not Recommended for routine AIS adjuvant treatment due to insufficient evidence quality and poor comprehensive performance.

All neuroprotective agents in this study are adjuvant drugs for AIS. Anisodamine has been reported for cerebrovascular diseases, but its evidence quality is insufficient for inclusion in this research; future studies can supplement its evaluation. This framework can guide hospital pharmacy committees and clinicians to select drugs rationally.

### 4.7 Other traditional Chinese medicine (TCM) agents

Ginkgolide Injection, Diterpene Ginkgolides Meglumine Injection and Ginkgo Biloba Leaves Injection are commonly used TCM injections for AIS in China. However, their pharmacological mechanisms are unclear, high-quality large-sample clinical verification is lacking, with total scores ranging from 45 to 57 points, and the overall evidence certainty is low, limiting their routine clinical application.

### 4.8 Limitations and future research recommendations

Although this study employed a systematic evaluation method, several limitations should be acknowledged: (Tu et al., 2023): Safety assessment relies primarily on package insert information and does not fully incorporate real-world pharmacovigilance data; (Zhu et al., 2025); Some efficacy scores are based on indirect comparison evidence and contain uncertainty; (GBD, 2021 Stroke Collaborators, 2023); The predictive validity of the scoring system has not been validated in external cohorts; (National Bureau of Statistics of China, 2023); There is no systematic inclusion of long-term outcome indicators. Future studies should incorporate real-world evidence, long-term follow-up data, and patient-reported outcomes (PROs) for more comprehensive evaluation.

Regarding the evaluation weights, we utilized a fixed-weight scoring system from a nationally recognized Chinese pharmaceutical guideline. While the allocation of points across dimensions (e.g., giving 28% weight to Efficacy and 27% to Safety) involves value judgments, these weights represent the expert consensus of clinical pharmacists and physicians across China rather than individual researcher preferences. By adopting this established methodology, the study provides a standardized and reproducible framework for evaluating AIS neuroprotective agents within the Chinese healthcare system (Zhao et al., 2024).

Geographic Specificity of Framework and Guidelines: Although this scoring system was developed based on guidelines and expert consensus, it has not yet undergone external validation. Future studies should validate the predictive capability of this scoring system through multi-center clinical data and real-world evidence. The evaluation is heavily based on a Chinese drug selection guide and clinical guidelines. This limitation affects the global generalizability of the findings in this study. For instance, both the AHA/ASA and ESO guidelines assign low evidence ratings or maintain a reserved stance toward the majority of the agents evaluated. Future research should incorporate a broader range of international clinical data to compare the therapeutic efficacy of neuroprotective agents across diverse ethnic backgrounds.

Heterogeneity of Evidence Quality: The quality and availability of evidence varied significantly among the ten drugs evaluated. This study has supplemented transparent methodological quality assessment and evidence certainty grading for all included studies, clearly clarifying the evidence limitations of each drug. High-scoring agents have relatively rigorous trial design and stable efficacy outcomes, while low-scoring agents have widespread limitations such as small sample size, single-center design and high bias risk. It is important to note that total score alone does not determine recommendation level. For final drug recommendations, we simultaneously considered (Tu et al., 2023): guideline recommendation levels (Zhu et al., 2025); strength of clinical evidence (GBD, 2021 Stroke Collaborators, 2023); completeness of safety data; and (National Bureau of Statistics of China, 2023) drug availability.

Regional Nature of Economic Data: The economic scoring in this study consists of two parts: 3 points for cost comparison with generic alternatives and 7 points for comparison with other competing therapeutic agents, mainly evaluating average daily treatment costs. This approach has limitations as it does not fully incorporate clinical outcome factors. However, in the field of neuroprotective agents, cost-effectiveness data remain relatively scarce. According to a systematic review of pharmacoeconomic studies in the Chinese acute ischemic stroke population, most existing studies still use modeling approaches, and reporting quality based on the CHEERS 2022 checklist requires improvement. Ideal pharmacoeconomic evaluation should incorporate incremental cost-effectiveness ratios (ICERs) or cost-effectiveness ratios (CERs), along with comprehensive indicators such as QALYs. However, limited by the sufficiency of currently reported literature, we adopted average daily cost as our preliminary economic assessment metric. Future studies should follow CHEERS 2022 standards and report more comprehensive cost-effectiveness data. The economic dimension of this evaluation relies on pricing data from a single regional procurement platform. We acknowledge that variations in provincial tender prices and local medical insurance reimbursement policies could influence the ‘Economy’ score. For instance, the actual out-of-pocket costs for patients may differ based on local Class B drug reimbursement ratios. Consequently, the economic rankings presented here should be interpreted as a methodological reference. Clinical decision-makers in other regions are encouraged to substitute these values with their localized bidding prices when applying this framework.

Dynamic Nature of the Research Field: The field of neuroprotection is rapidly evolving. In recent years, research on neuroprotection mechanisms has made significant progress, particularly studies on mitochondrial targeting and cellular apoptosis regulation (Li et al., 2019), which provide new molecular biological evidence for evaluating neuroprotective agent efficacy. Mitochondrial dysfunction is considered one of the core mechanisms of neuronal death post-stroke, and intervention strategies targeting this mechanism are becoming a research hotspot. The role of metabolic regulation in post-stroke recovery has also attracted widespread attention (Zhang et al., 2024). Energy metabolism reprogramming not only affects acute-phase neuronal survival but also participates in later neuroplasticity and functional recovery processes, providing deeper biological rationality support for the efficacy dimension of our multi-dimensional scoring system.

Research on neuroinflammation and ischemic brain injury mechanisms provides complementary perspectives for the drug evaluation framework. Research progress on apelin signaling pathway (Luo et al., 2022) highlights its important role in regulating blood-brain barrier permeability and neuroinflammatory responses, which may affect the effective delivery and efficacy manifestation of neuroprotective agents. Research on ROS-scavenging strategies (Shen et al., 2025) further emphasizes the position of oxidative stress in stroke pathogenesis, which is of important reference value for identifying adverse reactions in safety assessment. Advances in systems pharmacology and drug interaction modeling (Wang et al., 2025) provide a theoretical framework for understanding combination therapy of neuroprotective agents. Future research should explore multi-target synergistic effects based on this foundation and optimize dosing regimens.

For Traditional Chinese Medicine (TCM)-related agents, network pharmacology studies (Zhang et al., 2025; Zhou et al., 2025) suggest that TCM formulations may play comprehensive protective roles by regulating multiple ischemia-related pathways. This mechanistic understanding provides a theoretical basis for cross-cultural drug evaluation in our scoring system and also helps enhance the international relevance of this study.

This evaluation had a literature search cutoff date of Dec 31, 2025, and any significant clinical trials published after this date were not included, potentially impacting the timeliness of the conclusions. It is important to emphasize that the quantitative scoring system constructed in this study relies largely on reported information quality rather than robust clinical and economic evidence. Its use should be understood as an initial screening tool to assist clinical decision-making, rather than a deterministic recommendation basis. For reimbursement or national formulary inclusion decisions, comprehensive consideration of large-scale randomized controlled trials, real-world evidence, and pharmacoeconomic evaluation results is required. Future policy decisions should be based on pharmacoeconomic evaluations following CHEERS 2022 standards, combined with multi-center clinical data and cost-effectiveness analysis for more scientific decision-making.

This multi-dimensional evaluation identifies Butylphthalide and Sodium Chloride Injection as the strongly recommended and most robustly supported neuroprotective option, with Edaravone and Dexborneol Concentrated Solution for Injection and Urinary Kallidinogenase for Injection as weakly recommended alternative options for acute ischemic stroke within the Chinese clinical context. By reconciling pharmaceutical characteristics with localized economic and insurance priorities, this framework provides a scientific tool for hospital formulary committees to standardize adjuvant therapy and promote rational drug use. Ultimately, while these findings provide clear guidance for immediate clinical selection, they underscore the necessity for continuous evidence generation through large-scale trials and real-world studies to refine neuroprotective strategies and bridge the gap between regional clinical practices and international evidence standards.

## Data Availability

The original contributions presented in the study are included in the article/Supplementary Material, further inquiries can be directed to the corresponding author.
